# Redox-Responsive Heparin–Chlorambucil Conjugate Polymeric Prodrug for Improved Anti-Tumor Activity

**DOI:** 10.3390/polym12010043

**Published:** 2019-12-27

**Authors:** Abegaz Tizazu Andrgie, Yihenew Simegniew Birhan, Tefera Worku Mekonnen, Endiries Yibru Hanurry, Haile Fentahun Darge, Rong-Ho Lee, Hsiao-Ying Chou, Hsieh-Chih Tsai

**Affiliations:** 1 Graduate Institute of Applied Science and Technology, National Taiwan University of Science and Technology, Taipei 106, Taiwan; habegaz21@gmail.com (A.T.A.); yihenews@gmail.com (Y.S.B.); tefe16@gmail.com (T.W.M.); Endris_Yibru@dmu.edu.et (E.Y.H.); fentahunhailebdu@gmail.com (H.F.D.); wherelove8@gmail.com (H.-Y.C.); 2 Department of Chemical Engineering, National Chung Hsing University, Taichung 402, Taiwan; rhl@dragon.nchu.edu.tw; 3 Advanced Membrane Materials Center, National Taiwan University of Science and Technology, Taipei 106, Taiwan

**Keywords:** chlorambucil, combination chemotherapy, heparin, prodrug, self-assembly

## Abstract

Polymeric prodrug-based delivery systems have been extensively studied to find a better solution for the limitations of a single drug and to improve the therapeutic and pharmacodynamics properties of chemotherapeutic agents, which can lead to efficient therapy. In this study, redox-responsive disulfide bond-containing amphiphilic heparin–chlorambucil conjugated polymeric prodrugs were designed and synthesized to enhance anti-tumor activities of chlorambucil. The conjugated prodrug could be self-assembled to form spherical vesicles with 61.33% chlorambucil grafting efficiency. The cell viability test results showed that the prodrug was biocompatible with normal cells (HaCaT) and that it selectively killed tumor cells (HeLa cells). The uptake of prodrugs by HeLa cells increased with time. Therefore, the designed prodrugs can be a better alternative as delivery vehicles for the chlorambucil controlled release in cancer cells.

## 1. Introduction

Cancer is a group of diseases caused by an unusual proliferation of abnormal cells in the body, which is one of the leading causes of mortality and morbidity worldwide [1,2]. Several types of cancer treatments, which depend on the type and stage of tumor progression, are applied to kill cancer cells or inhibit their proliferation. Although chemotherapy is limited by poor pharmacokinetic properties, it is still the most commonly used strategy to treat different forms of cancer. Recent advances in nanotechnology have resulted in the fabrication of different nanocarriers for the effective delivery of therapeutics and thereby overcoming various limitations like rapid clearance, poor solubility, instability, multi-drug resistance, lack of disease targeting, and other side effects on normal tissues [3,4,5]. Among the different approaches explored so far, prodrug-based delivery systems have garnered considerable research attention due to their outstanding properties that can help to subdue the barriers of the traditional therapeutic delivery system [6,7,8]. Most bioactive hydrophilic polysaccharides have been directly conjugated to active drug molecules, which confers favorable characteristics to the resulting prodrugs, like improve solubility, non-toxicity, diminished immunogenicity, and proteolytic resistance.

Heparin is a highly anioic natural polysaccharide commonly used as an anticoagulant in clinical practice [9,10]. It is water-soluble and extensively studied for different biomedical applications. Heparin can bind with various growth factors like basic fibroblast growth factor and vascular endothelial growth factor [11]. Previously, we demonstrated that heparin is an efficient anti-metastatic agent. The anticoagulant properties of heparin are mainly due to its pentasaccharide sulfate groups of unique binding affinity to anti-thrombin (AT)-binding domains. The removal of these sulfate groups from heparin results in a dramatic drop in anticoagulant activity, but its anti-metastasis effect remains active [12]. Therefore, the use of heparin as the backbone for grafting the anti-cancer drugs may augment the effectiveness of anti-cancer drugs with its synergistic anti-metastatic activity. Chlorambucil (Chl) is an aryl nitrogen mustard-based DNA-alkylating anti-cancer drug that has been widely applied for different types of cancer diseases. Chlorambucil’s mechanism of action in cancer cells is binding its two reactive chloroethyl side chains with the nucleobases adenine and guanine at N3 and N7 that halt DNA replication and DNA damage through DNA strand linking [13,14,15]. Chlorambucil has a low water solubility, short half-life due to rapid degradation in the plasma, and serious toxicity to normal tissues, which are potential limitations in its clinical applications [16,17]. Therefore, strategies to improve the stability, solubility, and non-specific toxicity of Chl are highly required to improve its anti-cancer effect. Furthermore, in the nanocarrier drug-delivery system, almost all drug vehicles have non-therapeutic potency by themselves. In some cases, the degradation of carriers may cause systemic adverse effects. Recently, drug–drug conjugates have been investigated as a promising approach in delivery systems [8,18]. For example, Ping et al. developed a self-assembling amphiphilic drug by conjugating two hydrophilic–hydrophobic anti-cancer drugs [19]. The two drugs were conjugated with an ester bond, which is hydrolyzed to release free drugs.

Introducing a stimuli-sensitive linker between two bioactive molecules is an additional advantageous approach to upgrade the prodrug delivery system, which allows rapid drug release due to being triggered by specified stimuli [20,21]. A stimuli-responsive system is an extensively studied method to trigger the release of active drugs from prodrugs [22,23,24]. Interestingly, the tumor cell microenvironment has unique physiological characteristics that are crucial for the development of the stimuli-responsive system. Indeed, tumor cell microenvironment possess redox-, acidic-pH-, enzyme-, and reactive oxygen species (ROS)-responsive characteristics [22]. Redox-responsiveness is used for targeting tumor tissue due to the higher concentration of glutathione (GSH) and ROS, such as hydrogen peroxide (H_2_O_2_) in tumor intracellular compartment (~2–10 mM) than that in normal tissue (~2–20 µM) [25]. Designing redox-responsive drug carriers has garnered interest over the past years. A disulfide bond (-S-S-) is used as a redox-responsive linker, which is relatively stable in plasma and can be cleaved in a reductive environment; thereby enabling rapid release of the loaded drug [26,27].

**Scheme 1:**
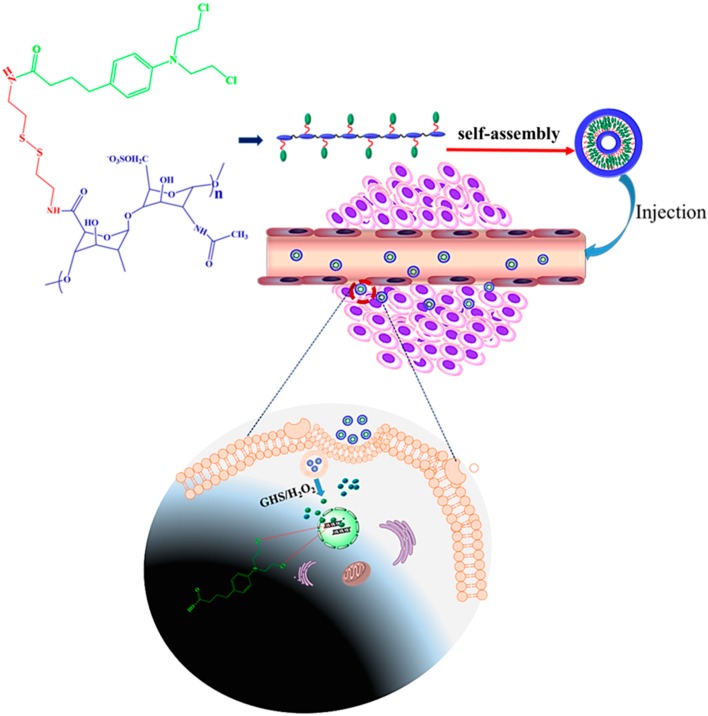
Schematic illustration of the Hep–Chl prodrug self-assembly and Chl intracellular drug release.

Here, water-soluble bioactive polysaccharide heparin was conjugated with Chl by a redox-sensitive disulfide bond-containing cystamine (Cys) as a linker, hereafter referred to as Hep–Chl. The release behavior of active drug Chl, as well as Hep, was based on redox triggering of -S-S- bond in the intracellular environment of cancer cells (Scheme 1). The heparin chain was grafted with Cys using the EDC/NHS coupling reaction. Then, Hep-Cys was further conjugated with poorly water-soluble Chl by applying a similar reaction, resulting in an amphiphilic polysaccharide-drug conjugate-based prodrug capable of self-assembly into spherical polymeric nanoparticle in aqueous system. The size and morphology of the self-assembled prodrugs and their redox stimulus-responsive release behaviors were studied. In vitro cellular viability and uptake of Hep–Chl were evaluated using cultured HaCaT and HeLa cells.

## 2. Experimental Section

### 2.1. Materials

Materials used for the fabrication of nanoparticles: Hep, Chl, and Cys were obtained from Sigma Aldrich (St. Louis, MO, USA). Catalytic chemical for reactions: Ethyl-3-[3-dimethylaminopropyl] carbodiimide hydrochloride (EDC) and N-hydroxysuccinimide (NHS) were obtained from Acros Organics (Geel, Belgium). For the experimental test: Solvents like 2-(N-morpholino) ethanesulfonic acid hydrate (MES) and PBS were also obtained from Sigma Aldrich (St. Louis, MO, USA). Cell room materials for MTT assay: Dulbecco’s modified Eagle medium (DMEM), trypsin, and penicillin were obtained from Gibco and 3-[4-dimethyl-2-thiazolyl]-2,5-diphenyl-2H-tetrazolium bromide (MTT) was purchased from Sigma Aldrich (St. Louis, MO, USA). Both HeLa and HaCaT cells were obtained from the BioResource collection and Research Center (Hsinchu, Taiwan). Deionized water was used in all the experiments, and it was obtained using a Millipore water purification system. All reagents and buffer solution components were of analytical grade.

### 2.2. Conjugation of Hep–Chl

The synthesis of disulfide bond cross-linked Hep–Chl was carried out using Cys as a redox labile linker. Briefly, Hep (200 mg) was dissolved in MES buffer solution (15 mL, 0.01 M), and then excess EDC and NHS (300 and 400 mg, respectively) were applied to the mixture and stirred at 25 °C. After 12 h, Cys was added under constant stirring and the reaction was carried out for an additional 24 h. Then, the reaction solution was dialyzed (MWCO 1 kDa) against distilled water for three days and finally lyophilized and kept at 10 °C until used in the subsequent reaction.

In another reaction, the carboxylic acid functional group of Chl was activated with excess EDC and NHS in DMSO solvent for 12 h. Finally, the heparin-cystamine (Hep-Cys) solution (in MES buffer solution) was added to activate Chl. The reaction was conducted for 24 h at 25 °C. The sample was dialyzed (MWCO 1 kDa) in water/DMSO at a ratio of 1:1 (v/v) for three days and lyophilized. By following the above procedure, three batches of heparin–Chl conjugates (Hep–Chl-1, Hep–Chl-2, and Hep–Chl-3) were prepared with different grafting ratios of Chl to the heparin backbone.

### 2.3. Preparation of the Hep–Chl Prodrug Self-Assembled Nanoparticle

To prepare the Hep–Chl self-assembled prodrug nanoparticle, the conjugates were dissolved in DMSO by gentle heating, and then added into water dropwise. The mixture was stirred overnight and dialyzed (MWCO 1 kDa cutoff) against distilled water at room temperature for 2 days to remove DMSO. Then, an aqueous suspension containing the self-assembled Hep–Chl nanoparticles was obtained.

### 2.4. Characterization of the Hep–Chl Prodrugs

The synthesis of Hep–Chl was confirmed by proton nuclear magnetic resonance (^1^H NMR) spectroscopy (Bruker AVANCE 500.163 MHz), using D_2_O as a solvent and UV-vis spectrophotometer (JASCO-V-650). Raman spectroscopy was also performed using a JASCONRS-5100 Laser Raman Spectrometer. Infrared (IR) spectra were obtained using an attenuated total reflectance (ATR) spectroscope (JASCO, ATR-FTIR-6700). Hydrodynamic mean diameter and size distribution of the Hep–Chl self-assembled nanoparticles were measured at 25 °C using a dynamic light scattering (DLS) system (Horiba Zeta sizer-100 system; Malvern Instruments, Malvern, UK). The stability of the Hep–Chl prodrugs nanoparticles was examined in human serum albumin (HSA). Briefly, the Hep–Chl prodrug nanoparticles (0.5 mg/mL) in PBS were incubated in 2.5 mL HSA aqueous media at 37 °C for 1, 2, 4, 8, 16, 24, 48, and 72 h time intervals and then the size of nanoparticles was measured with DLS. Field-emission scanning electron microscopy (FESEM) (JSM-6500F; JEOL) and atomic force microscopy (AFM NX10; AFM Park systems, Suwon, South Korea) were also performed to observe the morphology and to estimate the size of the Hep–Chl nanoparticles after the sample solutions were dried on a silicon substrate.

### 2.5. Drug Release in the Simulated Redox Environment

The in vitro Chl release profile of the self-assembled Hep–Chl prodrug was studied in the dialysis membrane in PBS buffer (pH 7.4) containing 0.1% H_2_O_2_ and 6 mM GSH at 37 °C, respectively. Briefly, 1 mL of Hep–Chl prodrug solution was transferred into a dialysis tube (cutoff molecule weight 1000 Da) and immersed in vials containing 6 mM GSH and 0.1% H_2_O_2_ in 15 mL PBS, followed by stirring at 37 °C to mimic physiological environment. At predetermined time (0.5, 1,3, 6, 9, 12, 24, 48, and 72 h), 3 mL aliquots from the dialysate was sampled to quantify Chl released. For each 3 mL of aliquot sampled, an equal volume of fresh PBS solution (with or without stimuli) was replaced. The amount of Chl in the release media was estimated using a UV-vis (JASCO V-730, Oklahoma, USA) spectrophotometer at 304 nm. The cumulative release of the drug was calculated as the total percentage of Chl released from the dialysis membrane. The data are presented as an average of triplicate measurements.

### 2.6. Cell Viability Assay

Cellular viability test was examined using the MTT assay with HeLa, HaCaT, and RAW264.7 cells. Both HeLa and HaCaT cells (1 × 104 cell/well) were cultured in DMEM containing 10% (v/v) FBS, 1% (w/v) sodium pyruvate, and 1% (w/v) streptomycin at 37 °C under a humidified atmosphere with 5% CO_2_ for 24 h. The media was discarded, the cells were treated with serial dilutions of Hep–Chl prodrug (0, 0.2, 0.4, 0.6, 0.8, and 1 mg/mL) for 24 h. The RAW264.7 and HeLa cells were further treated for 72 h with Chl alone (0, 0.2, 0.4, 0.6, 0.8, and 1 mg/mL) and Hep–Chl (mg/mL equivalent of Chl) for long term treatment evaluation. After treatment for each specific time, cells were washed three times and incubated with 20 µL of MTT (5 mg/mL) solution at 37 °C for 4 h. The MTT solution was removed and 100 μL of dimethyl sulfoxide (DMSO) was added to dissolve the formazan crystals. The absorbance of the sample was measured using an enzyme-linked immunosorbent assay (ELISA) reader (Thermo Multiskan FC microplate photometer; Thermo Fisher Scientific, Waltham, MA, USA) at 570 nm. Cell viability (%) was calculated using Equation (1).

(1) $$Cell\;viability\;\left(\%\right)\;=\;\frac{\mathrm{Absorbance}\;\mathrm{of}\;\mathrm{test}}{\mathrm{Absorbance}\;\mathrm{of}\;\mathrm{control}}\;\times \;100.$$

For further evaluation of the pharmacological activity and the cytotoxic effect of Hep–Chl on cancer cells, a live/dead cell staining test was performed to visualize the cell viability by fluorescence microscopy. HeLa cells were cultured with Hep–Chl and Chl with an equivalent in Chl concentration for 12 h. Afterward, the cells were washed with PBS (pH = 7.4) and the solution of calcein acetoxymethyl ester (2 µm, calcein-AM) and propidium iodide (4 µm, PI) was added to the cells. The cells were then incubated for 30 min at 37 °C and 5% CO_2_. Next, they were washed thrice with PBS and then imaged with fluorescence microscopy.

### 2.7. Examination of In Vitro Cellular Uptake of Hep–Chl Nanoparticles

To study cellular internalization of the Hep–Chl nanoparticles, Rhodamine B was encapsulated with nanoparticles and used as a marker. The Hep–Chl conjugates were dissolved in DMSO by gently heating, and then added into aqueous Rhodamine B solution dropwise. The mixture was stirred overnight and dialyzed (MWCO 1 kDa cutoff) against distilled water at room temperature for 2 days to remove unbound Rhodamine B and DMSO. Then, an aqueous suspension containing Rhodamine B loaded with the self-assembled Hep–Chl nanoparticles was obtained, which were used to estimate cellular uptake. HeLa cells were seeded into a confocal dish at a density of 10 [5] cells/well and incubated at 37 °C under a humidified atmosphere with 5% CO_2_ overnight. The cells were treated with 0.8 mg/mL in medium and incubated for 3, 6, and 12 h. The culture media was discarded, and the cells were treated with DAPI (10 mg/mL) for 15 min for nucleus staining. Paraformaldehyde was used to fix the cells for 10 min at a temperature of 4 °C. Finally, the cells were imaged by confocal laser scanning microscopy (CLSM, Gyeonggi-do, South Korea).

## 3. Results and Discussion

### 3.1. Synthesis of the Hep–Chl Prodrug

**Figure 1:**
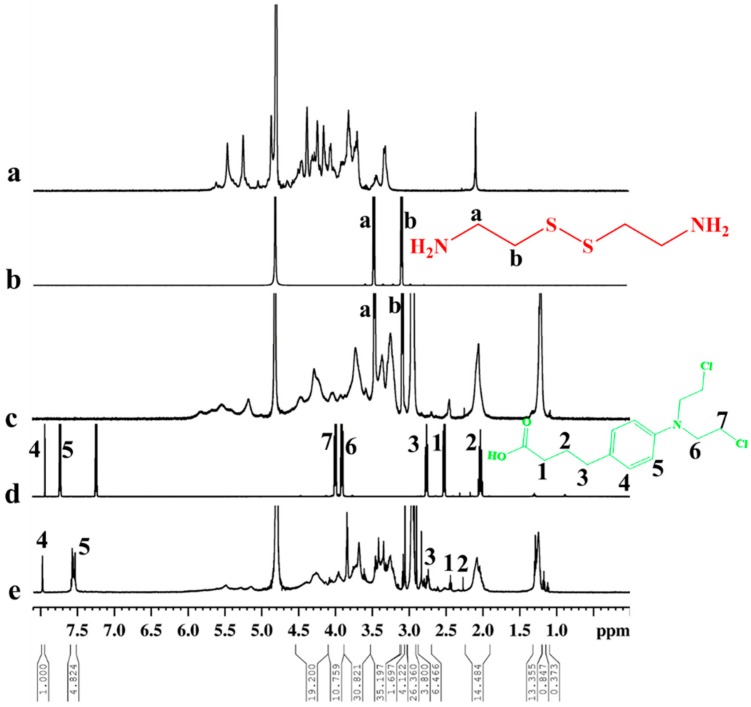
^1^H NMR spectra of Hep (**a**), cystamine (Cys) (**b**), Hep-Cys (**c**), Chl (**d**), and Hep–Chl (**e**) conjugate. CDCl_3_ is used as a solvent for Chl and D_2_O for others.

Recently, polymer-drug conjugate prodrugs have been synthesized as therapeutic agents to enhance drug stability and therapeutic efficiency. In this study, a dual-acting Hep–Chl prodrug was synthesized to investigate its in vitro anti-cancer activities. As depicted in Figure 1, the characteristic peaks of Cys in Hep-Cys appeared at δ = 3.5 (a) and δ = 3.0 ppm (b) without any shift, which confirmed the conjugation of Hep and Cys. Similarly, the peaks at δ = 2.5 (1), δ = 2.1 (2), δ = 2.7 (3), δ = 8 (4), and δ = 7.7 ppm (5) correspond to the protons of Chl, which did not present a clear chemical shift compared with the corresponding spectrum peaks of Hep–Chl. These labeled ^1^H NMR spectrum peaks indicated the successful conjugation of Hep with Chl.

**Figure 2:**
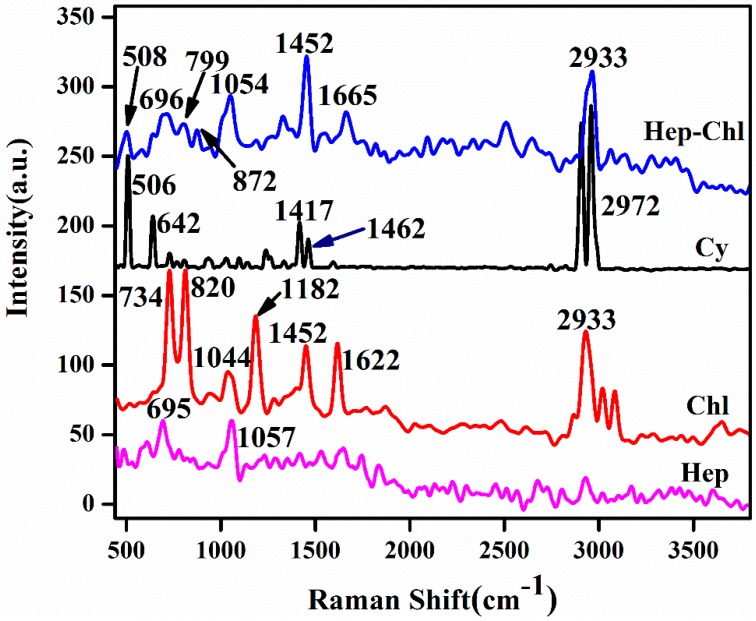
Raman spectra of heparin (Hep), chlorambucil (Chl), cystamine dihydrochloride (Cy), and the conjugate Hep–Cy–Chl.

The structure of Hep–Chl was further confirmed by Raman spectroscopy. Figure 2 shows the Raman spectra of the starting materials Hep, Cys, and Chl, and the conjugated copolymer Hep–Chl. The major Raman spectrum peaks of Hep were observed at 695 and 1057 cm^−1^, which were assigned to C-C-O stretching and O-S-O_3_^−^ vibration. The Raman spectrum of Cys presented very intense bands at 506, 642, 1417, and 2971 cm^−^^1^, which are associated with S-S, C-S, CH_2_, and asymmetric stretching of C-H, respectively [28]. The major Raman peaks of Chl were also observed at 734 (C-Cl), 820 (C-N-C), 1044 (C-C-H), 1182 (Ring), 1452 (ring C-C symmetric stretching), 1622 (C=O mixed NH), and 2933 cm^−1^ (CH). The Raman spectrum of the Hep–Chl prodrug showed the major peaks of the starting materials. It revealed O-S-O_3_^−^ vibration peaks of Hep at 1054 cm^−^^1^ and S-S peaks of Cys at 508 cm^−^^1^. Similarly, the characteristic Raman peak of Chl produced by C-Cl was observed at 696 cm^−^^1^. Therefore, the presence of these major bands of the starting materials, O-S-O_3_^−^ vibration peak of Hep, S-S peak of Cys, and C-Cl peak of Chl, proved the successful synthesis of Hep–Chl [21]. The bond formation between Hep-Cys and Chl was determined by FT-IR. In the FT-IR spectra (Figure S1), the new characteristic peaks of Hep–Chl were observed at 1778, 1620, and 1646 cm^−^^1^ in comparison with free Hep-Cys and Chl, which are associated with the stretching vibration of -C=O, and amide I and II bands, respectively [28,29]. These results further confirmed the conjugation of Hep-Cys with Chl.

**Figure 3:**
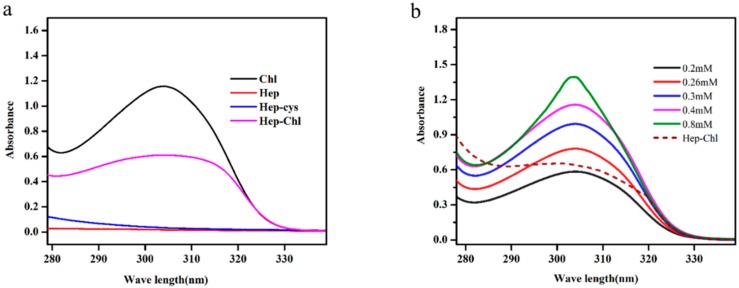
(**a**) UV-vis spectra of Hep, Hep-Cys, Chl, and Hep–Chl. (**b**) The concentration of Chl absorbance for the calibration curve at 304 nm.

The conjugation of Hep-Cys with Chl was also confirmed by UV-vis spectrophotometry [30,31,32]. As presented in Figure 3a, Chl was successfully grafted onto the Hep backbone, as shown by the UV absorption peak around 304 nm. There was no UV absorption peak for Hep and the Hep-Cys solution in the wavelength range of 275–400 nm. The amount of Chl coupled on the backbone of Hep was estimated by using the free Chl standard curve at 304 nm, obtained by UV-vis spectrophotometry (Figure 3b). The concentration of Chl was 0.33 mM, which suggests that around 61.33% of Chl was conjugated on Hep (Table 1). The UV–vis and ^1^H NMR spectra results indicated that Chl was successfully conjugated on Hep.

### 3.2. Formation of the Self-Assembled Prodrug

**Figure 4:**
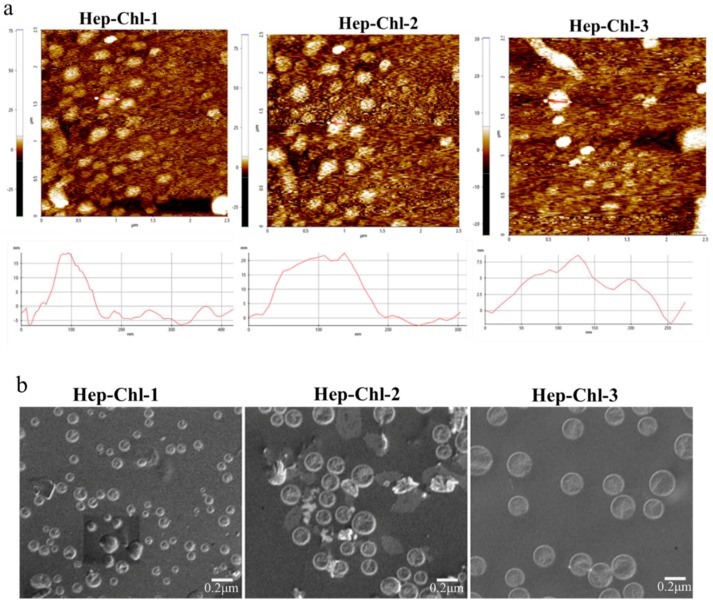

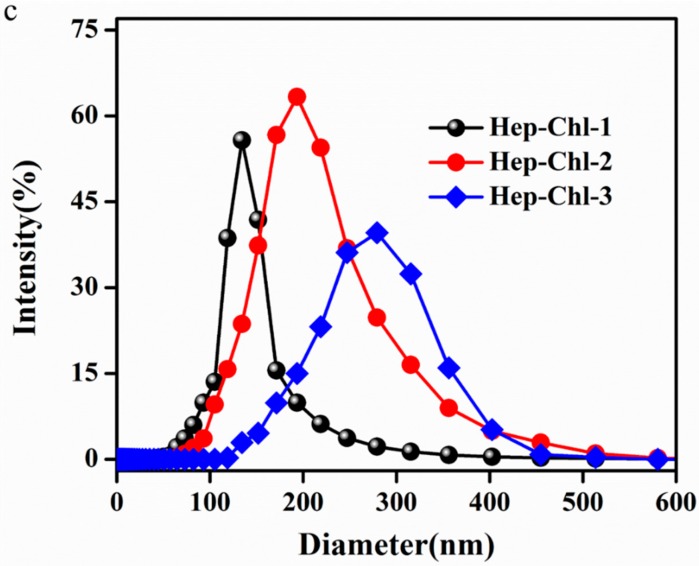
Particle morphology and size: (**a**) AFM images, (**b**) SEM images, and (**c**) particle size distribution Hep–Chl-1, Hep–Chl-2, and Hep–Chl-3 self-assembled prodrugs.

Amphiphilic polymers with suitable hydrophilic–hydrophobic balance could self-assemble into nanoparticles in an aqueous solution [33]. In the present study, the hydrophilic polysaccharide Hep and hydrophobic Chl were conjugated using Cys as a linker to form an amphiphilic Hep–Chl prodrug, which could easily self-assemble into nanoparticles in PBS solution. To confirm the formation of self-assembled Hep–Chl prodrug nanoparticles, DLS, SEM, and AFM were performed. The results of SEM and AFM revealed that the self-assembled Hep–Chl prodrug nanoparticles were spherical in all the conjugate samples (Figure 4a,b). Moreover, using the AFM images, we roughly estimated the particle size of Hep–Chl prodrugs: Hep–Chl-1 ~ 140 nm, Hep–Chl-2 ~ 180 nm, and Hep–Chl-3 ~ 255 nm. After treatment with 6 mM GSH and 0.1% H_2_O_2_, the AFM images revealed that the Hep–Chl-1 prodrug nanoparticles appeared irregularly shaped and larger. As shown in Figure S2, the reduction and oxidation of disulfide bonds by GSH and H_2_O_2_ disrupted the nanoparticles that induced an increase in size and irregular shape. From the DLS measurements, the mean hydrodynamic diameter of the nanoparticles was estimated to be 150 nm for Hep–Chl-1 prodrug, 210 nm for Hep–Chl-2 prodrug, and 300 nm for Hep–Chl-3 prodrug (Figure 4c). As the concentration of Chl increased, more Chl molecules were distributed in the prodrug bilayer, resulting in an increase in the particle size [34]. The size of each prodrug nanoparticle determined from AFM data was smaller than that from the DLS data [21,35]. As samples for AFM measurement were dried on silica waiver, the nanoparticles were estimated to be smaller; whereas, for DLS measurement, the diameter of the hydrated layer is considered and therefore the nanoparticles were bigger. From the three batches, the smallest prodrug Hep–Chl-1 was selected for further investigation (in vitro cell viability and cellular uptake studies). The stability of nanoparticle in aqueous media with 50 mg/mL human serum albumin (HSA) was examined. The normal concentration of HSA in human blood serum is 35–50 mg/mL. The Hep–Chl-1 prodrug nanoparticles (0.5 mg/mL) in PBS was incubated in 2.5 mL HSA aqueous media at 37 °C for 1, 2, 4, 8, 16, 24, 48, and 72 h time intervals. The result showed that there was no significant difference between the sizes of nanoparticles in serum protein and in PBS without serum protein (Figure S3). This indicated that Hep–Chl-1 prodrugs nanoparticles are stable in human blood and could be useful for in vivo applications.

### 3.3. In Vitro Drug Release Studies

**Figure 5:**
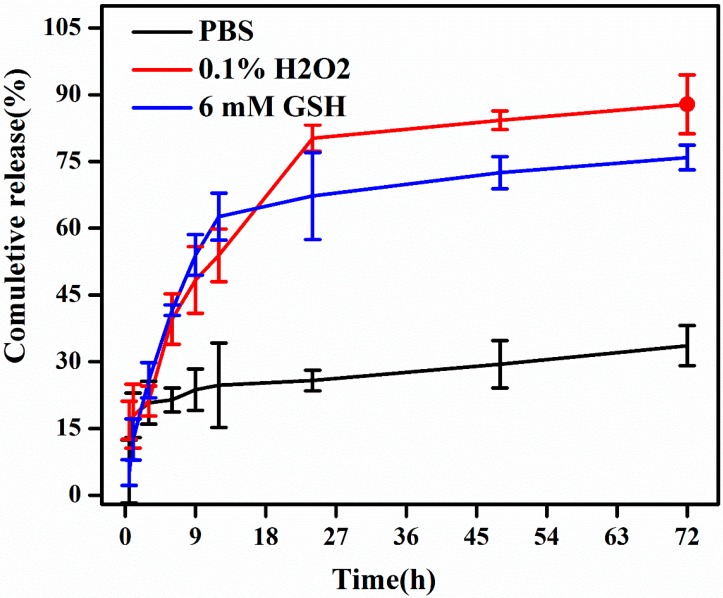
Redox-responsive drug release profile of Hep–Chl self-assembled prodrug nanoparticles in PBS only, 2 mg/mL GSH/PBS, and 0.1% H_2_O_2_ PBS.

The redox-responsive release profile of Chl from the Hep–Chl prodrug nanoparticle was investigated using the dialysis method under simulated physiological conditions. For comparison, the release profile of Chl under normal physiological conditions (in PBS without any stimuli) was also studied. The prodrugs were incubated with cancer cells mimicking intracellular environment in PBS solution (pH 7.4) with 0.1% H_2_O_2_ and 6 mM GSH at 37 °C. The in vitro Chl cumulative release profile from the Hep–Chl prodrug nanoparticles is presented in Figure 5. Under normal conditions without any stimuli, the cumulative release of Chl was below 30% within 72 h, suggesting that the release of Chl from the prodrugs without redox stimuli was below 30%. However, the cumulative release of Chl in the redox stimuli in PBS containing 6 mM GSH and 0.1% H_2_O_2_ was found to be faster, and 75% and 85% of Chl was released, respectively, within 72 h. Both bioactive drugs, Hep and Chl, were covalently linked to each other by disulfide bonds, which could be cleaved to activate Hep and Chl through a reducing agent GSH and an oxidizing agent H_2_O_2_ [36,37,38]. Overall, this stimuli-responsive prodrug-based delivery system could facilitate the accumulation of drugs in tumor tissues and improve cancer therapeutic efficacy [14].

### 3.4. In Vitro Cytotoxicity Study

**Figure 6:**
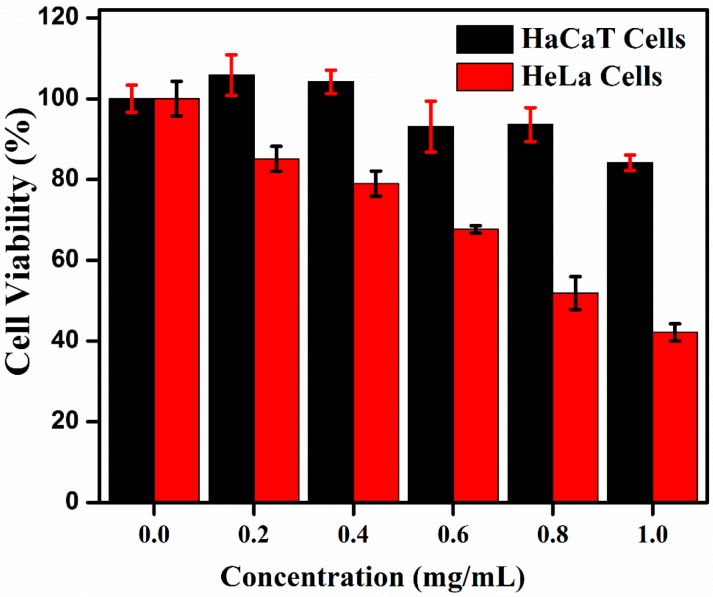
Cell viability of HeLa (red) and HaCaT (black) cells treated with Hep–Chl for 24 h. Cell viability was measured using the standard MTT assay. Data are presented as mean ± standard deviation.

**Figure 7:**
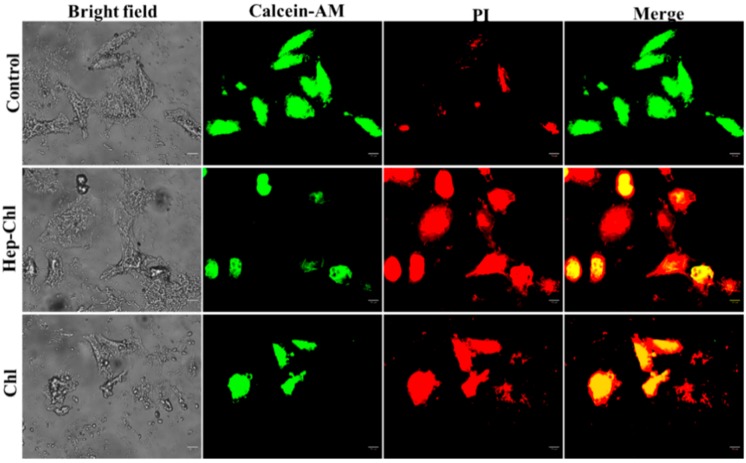
Cell viability. Fluorescence images of the HeLa cells stained with calcein-acetoxymethyl ester (AM)/propidium iodide (PI) with Hep–Chl, Chl, and PBS (control).

To evaluate the in vitro biocompatibility and pharmacological activity of the Hep–Chl prodrugs, the viability of HeLa and HaCaT cells treated with the prodrugs was examined using the MTT assay. The safety of prodrug was determined after 24 and 72 h of incubation with a series of Hep–Chl concentrations (0, 0.2, 0.4, 0.6, 0.8, and 1 mg/mL equivalent concentration of Chl), which revealed no potential cytotoxicity of the Hep–Chl prodrug nanoparticles against HaCaT and RAW264.7 cells at a maximum dose of 1 mg/mL. This may be attributed to the lower level of GSH and H_2_O_2_ and thereby insufficient cleavage of Chl from the Hep–Chl prodrug in normal cells [37,38,39,40] (Figure 6). In contrast, the viability of HeLa cells treated with Hep–Chl prodrug nanoparticles with the same concentrations was decreased to 41% at 24 h and 23% at 72 h treatments with increase in concentration (Figure S4a), which was considerably lower than that of RAW264.7 cells treated with the same concentration of the prodrug (Figure S4b). The concentration of GSH and H_2_O_2_ in tumor cells was considerably higher than that in normal cells [41,42,43,44], which cleaved disulfide linkage of the Hep–Chl prodrug resulting in a significant increase in the release of Chl within tumor cells, causing pronounced cell death. In addition, live/dead cell staining was used to visualize the cell viability. Live and dead cells were stained with Calcein-AM and propidium iodide (PI), respectively. In the fluorescence image results shown in Figure 7, compared with the control group (treated with PBS), the viability of the live cells of HeLa cells treated with Hep–Chl and free Chl was significantly reduced. Moreover, free Hep could help reduce cancer cell metastasis as reported in our previous study [12]. Therefore, the Hep–Chl prodrug nanoparticles selectively killed more tumor cells than the normal cells because of the high redox potential gradient between the tumor and normal cells [20,21].

### 3.5. In Vitro Cellular Uptake of Hep–Chl Nanoparticles

**Figure 8:**
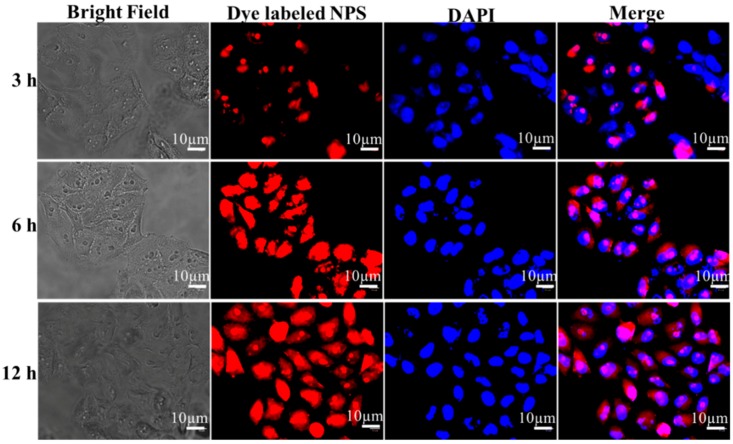
Cellular uptake of prodrug nanoparticles. The fluorescence intensity measuring Rhodamine B-labeled Hep–Chl uptake after 3, 6, and 12 h of treatment of HeLa cells.

In vitro cellular uptake of Hep–Chl was also confirmed by detecting fluorescence emitted by Rhodamine B dye loaded into the nanoparticles by fluorescence microscopy [45,46,47] (Figure S5). HeLa cells were incubated with Rhodamine B-loaded Hep–Chl nanoparticles for 3, 6, and 12 h. As shown in Figure 8, the Rhodamine B-loaded Hep–Chl-treated cells displayed intense red fluorescence in the cytosol and nucleus, which indicates the presence of nanoparticles inside the cells and successful prodrug delivery. Cellular uptake of Hep–Chl prodrugs in HeLa cells was increased in a time-dependent manner.

## 4. Conclusions

In this study, amphiphilic Hep–Chl prodrug nanoparticles were fabricated and their in vitro anti-cancer activity was investigated against HeLa and HaCaT cells. Hep was conjugated with Chl through a disulfide linkage to overcome the limitations of Chl. This Hep–Chl prodrug could self-assemble into spherical nanoparticles in the aqueous system, which exhibited a sustained release of active drug owing to its redox-responsive behavior. Rhodamine B-labeled nanoparticle accumulation in the cytosol of HeLa cells confirmed the uptake of Hep–Chl prodrugs. We confirmed that the Hep–Chl prodrug nanoparticles selectively killed tumor cells due to high redox potential in the tumor cells. In summary, co-delivery of Hep and Chl via a redox-responsive prodrug approach could be a promising strategy to achieve a dual effect in cancer therapy.

## Figures and Tables

**Table 1:** Coupling reaction of heparin-chlorambucil (Hep-Chl).

Conjugate Prodrug	Feed Chl (mM)	Conjugated Chl (mM)	Yield (%)
Hep–Chl-1	0.36	0.27	75
Hep–Chl-2	0.53	0.31	60
Hep–Chl-3	0.65	0.32	49

## Supplementary Materials

The following are available online at https://www.mdpi.com/2073-4360/12/1/43/s1, Figure S1: FTIR spectrum of heparin (black), PNIPAm-NH_2_ (red), and conjugation (copolymer) (blue), Figure S2: Particle morphology by AFM imaging of Hep-Chl-1 prodrug nanoparticles and after treatment either with 6 mM GSH and 0.1% H_2_O_2_, Figure S3: The size of Hep-Chl-1 prodrug nanoparticles after incubating in human serum albumin and PBS with 1, 2, 4, 8, 16, 24, 48, and 72 h at 37 °C, Figure S4: MTT profiles of (a) HeLa cells and (b) RAW264.7 cells treated with Chl and Hep-Chl (equivalent amount of Chl) for 24 and 72 h, Figure S5: The fluorescence intensity measuring Rhodamine B labeled Hep-Chl-1 prodrug nanoparticle.
