# Supplementary material for: Redox-Responsive Heparin–Chlorambucil Conjugate Polymeric Prodrug for Improved Anti-Tumor Activity
Source: Polymers (Basel). 2019 Dec 27;12(1):43. doi: 10.3390/polym12010043 (PMC7023610; doi:10.3390/polym12010043)
Supplement: Supplementary file 1 [file polymers-12-00043-s001.pdf]

polymers MDPI

Supporting information

Redox-Responsive Heparin–Chlorambucil Conjugate Polymeric Prodrug for Improved Anti-Tumor Activity

Abegaz Tizazu Andrgie [1], Yihenew Simegniew Birhan [1], Tefera Worku Mekonnen [1], Endiries Yibru Hanurry [1], Haile Fentahun Darge [1], Rong-Ho Lee [2], Hsiao-Ying Chou [1] and Hsieh-Chih Tsai [1,3,*]

1. Graduate Institute of Applied Science and Technology, National Taiwan University of Science and Technology, Taipei 106, Taiwan, ROC; habegaz21@gmail.com (A.T.A.); yihenews@gmail.com (Y.S.B.); tefe16@gmail.com (T.W.M.); Endris_Yibru@dmu.edu.et (E.Y.H.); fentahunhailebdu@gmail.com (H.F.D.); wherelove8@gmail.com (H.Y.C.)
2. Department of Chemical Engineering, National Chung Hsing University, Taichung 402, Taiwan; rhl@dragon.nchu.edu.tw
3. Advanced Membrane Materials Center, National Taiwan University of Science and Technology, Taipei 106, Taiwan

* Correspondence: h.c.tsai@mail.ntust.edu.tw; Tel.: +886-2-27303625; Fax: +886-2-27303733

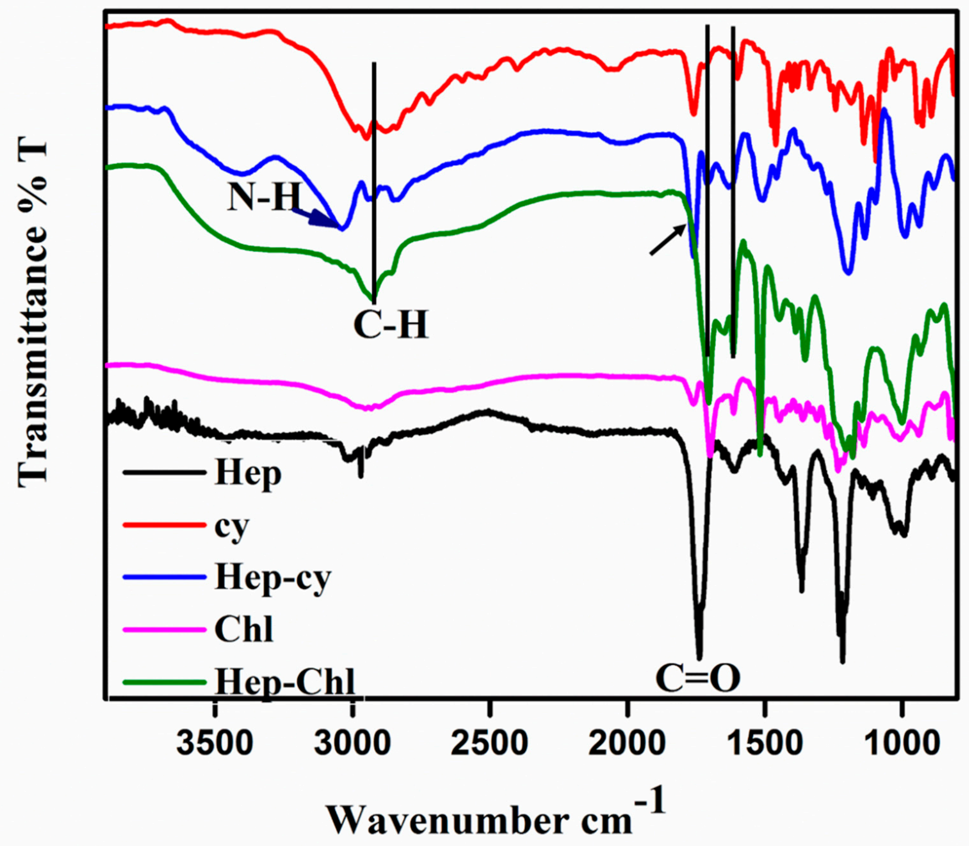

Figure S1. FTIR spectrum of heparin (black), PNIPAm-NH_2 (red), and conjugation (copolymer) (blue).

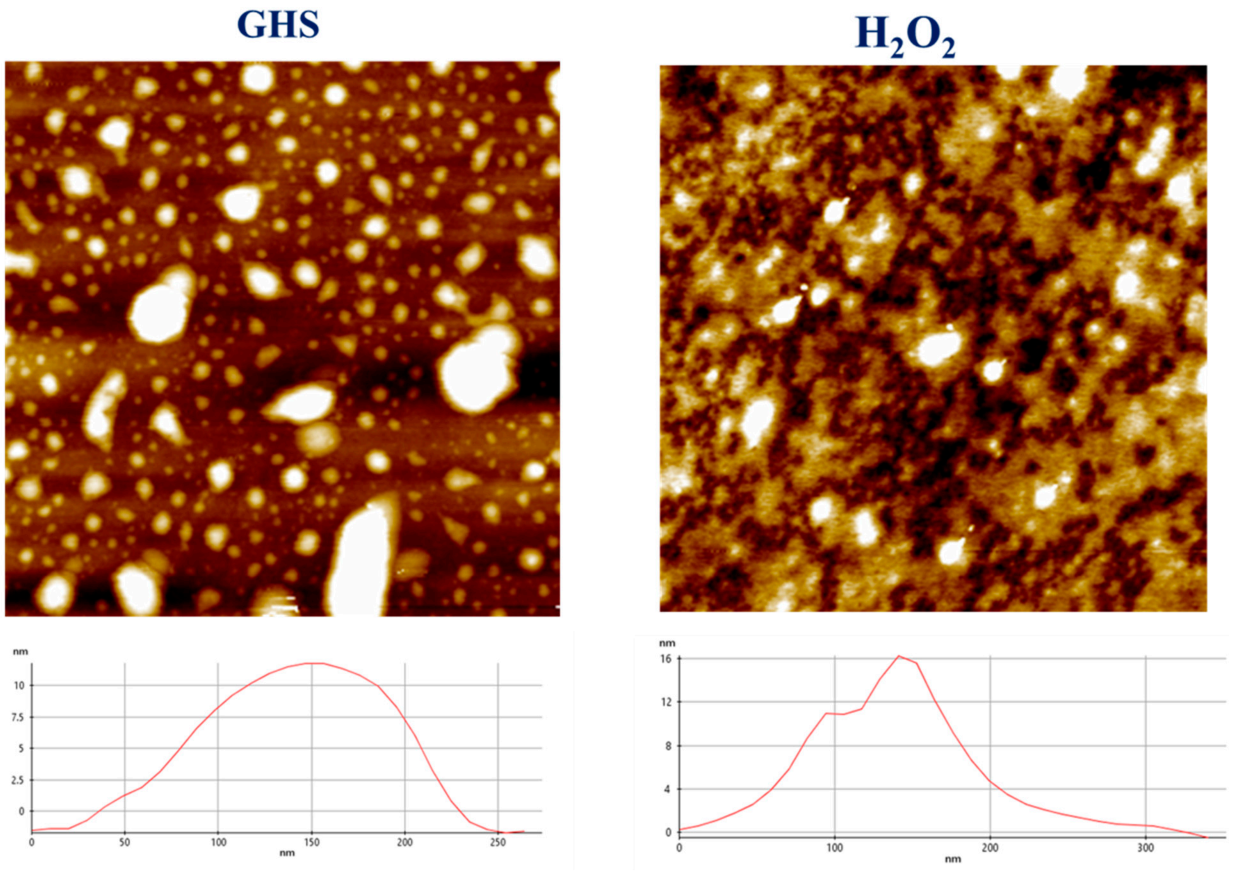

Figure S2. Particle morphology by AFM imaging of Hep-Chl-1 prodrug nanoparticles and after treatment either with 6 mM GSH and 0.1% H_2O_2.

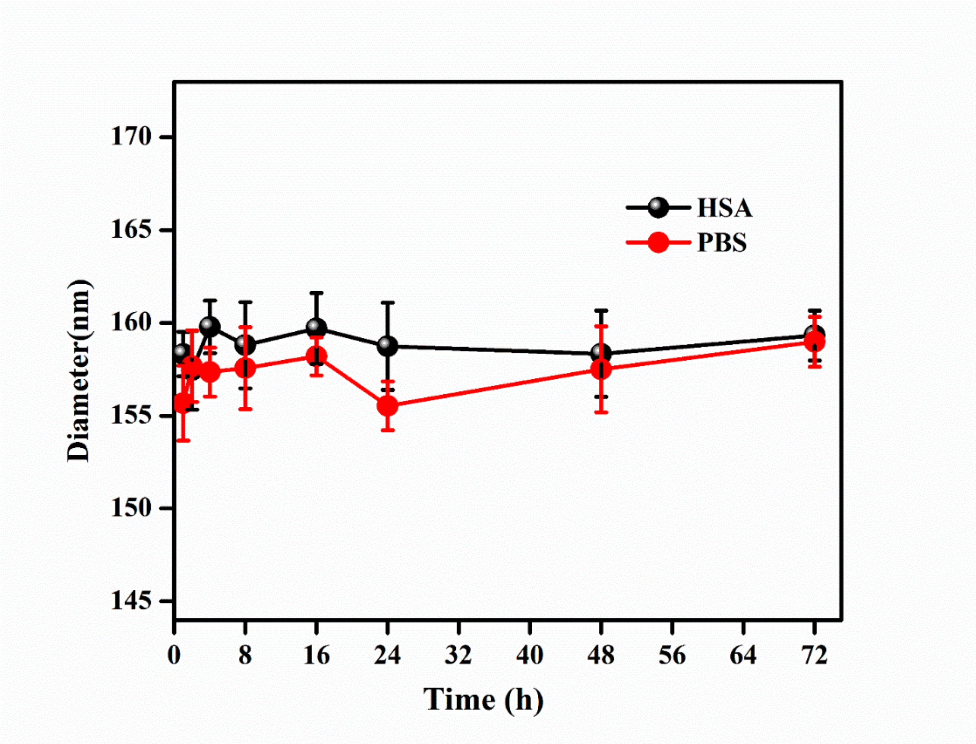

Figure S3. The size of Hep-Chl-1 prodrug nanoparticles after incubating in human serum albumin and PBS with 1, 2, 4, 8, 16, 24, 48, and 72 h at 37 ℃.

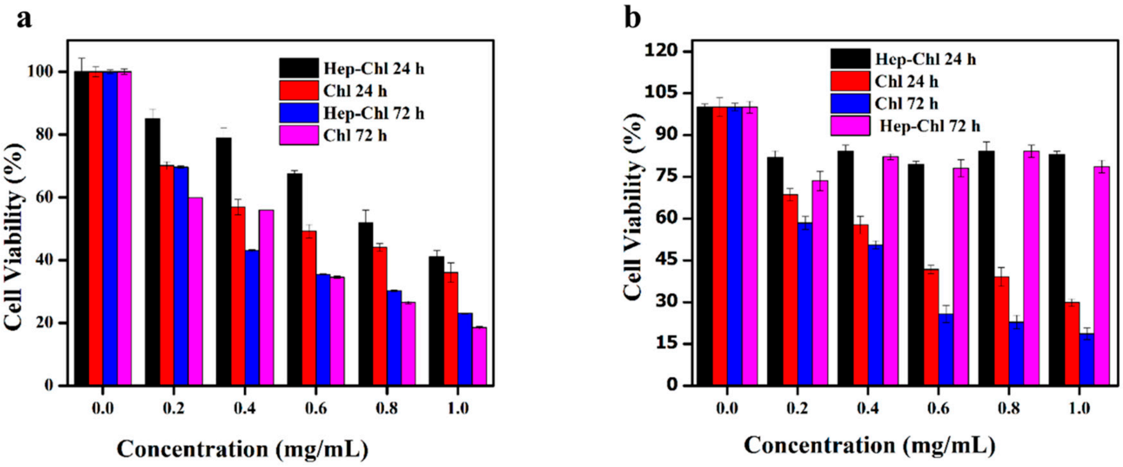

Figure S4. MTT profiles of (a) HeLa cells and (b) RAW264.7 cells treated with Chl and Hep-Chl (equivalent amount of Chl) for 24 and 72 h.

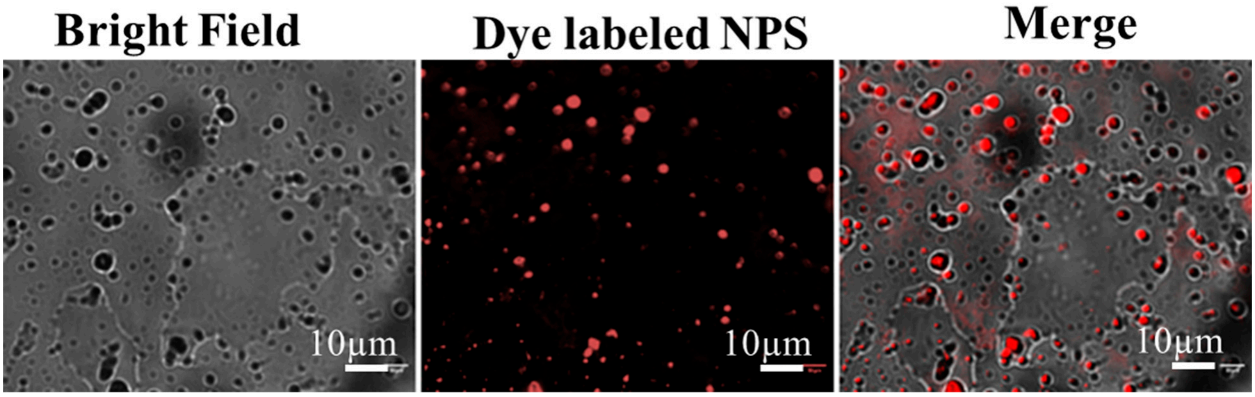

Figure S5. The fluorescence intensity measuring Rhodamine B labeled Hep-Chl-1 prodrug nanoparticle.
